# HIV-positive migrants’ encounters with the Swedish health care system

**DOI:** 10.3402/gha.v9.31753

**Published:** 2016-11-28

**Authors:** Manijeh Mehdiyar, Rune Andersson, Katarina Hjelm, Lene Povlsen

**Affiliations:** 1 Department of Infectious Diseases, Institute of Biomedicine, The Sahlgrenska Academy, University of Gothenburg and Sahlgrenska University Hospital, Gothenburg, Sweden; 2 Department of Social and Welfare studies, Linköping University, Campus Norrköping, Norrköping, Sweden; 3 Unit for Health Promotion Research, University of Southern Denmark, Esbjerg, Denmark

**Keywords:** HIV-positive, migrants, health care system, Sweden, Grounded Theory

## Abstract

**Background:**

There is limited knowledge about human immunodeficiency virus (HIV)-positive migrants and their experiences in the Swedish health care system. It is necessary to increase our knowledge in this field to improve the quality of care and social support for this vulnerable group of patients.

**Objective:**

The aim of this study was to describe the experiences of HIV-positive migrants and their encounters with the health care system in Sweden.

**Design:**

This is a Grounded Theory study based on qualitative interviews with 14 HIV-positive migrants living in Sweden, aged 29–55 years.

**Results:**

‘A hybrid of access and adversity’ was identified as the core category of the study. Three additional categories were ‘appreciation of free access to treatment’, ‘the impact of the Swedish Disease Act on everyday life’, and ‘encountering discrimination in the general health care system’. The main finding indicated that participants experienced frustration and discrimination because they were required to provide sexual partners with information about their HIV status, which is compulsory under the Swedish Disease Act. The study also showed that the bias or fear regarding HIV infection among general health care professionals outside of the infectious diseases clinics limited the access to the general health care system for HIV-positive migrants.

**Conclusions:**

The HIV-positive migrants appreciated the free access to antiviral therapy, but wished to have more time for patient–physician communications. The participants of this study felt discrimination in health care settings outside of the infectious diseases clinics. There is a need to reduce the discrimination in general health care services and to optimize the social support system and social network of this vulnerable group.

## Introduction

Migration has global impacts on health, especially affecting the human immunodeficiency virus (HIV) epidemic. There are a variety of obstacles to HIV care for migrants in Western Europe, including legal frameworks, immigration policies, and migrants’ social circumstances (1). This indicates that there is a clear need for more individualized support of this vulnerable population. Approximately, half of the 7,000 HIV-positive persons living in Sweden are migrants (2). The majority of this group is heterosexual and originates primarily from sub-Saharan Africa, Southeast Asia, and South America (2).

The care, treatment, and prevention of HIV/AIDS are influenced by a wide range of social, cultural, and political factors (3). For example, the low rate of HIV testing and an unwillingness to accept medical care among sub-Saharan migrants in Western Europe were found to be based on the fear of death and disease, as well as on the fear of stigma and discrimination (4). A study on undocumented Latino Americans in the United States reported that the late uptake of medical care among HIV-positive Latinos was related to the fear of deportation and the stigma attached to those who were HIV-positive within the family and the broader Latino community (5). Barriers to testing and care among African migrants in the United Kingdom and the Netherlands also were grounded in the fear of death and disease, and the fear of stigma and discrimination in their communities (6).

A study conducted in Sweden among HIV-positive people with African background identified a positive correlation between access to counseling, social networking, and openness about HIV status (7). A systematic review focusing on barriers to HIV testing, counseling, and care in Europe reported that a positive attitude of health care providers and an increased knowledge of the perceptions of patients are critical to improve the effectiveness of HIV testing and care (8). Despite the fact that socio-cultural diversity is increasing in the Swedish population, there are few studies focusing on cross-cultural diversity in the health care setting (9). In particular, not enough is known about the experiences of HIV-positive migrants within the Swedish health care system.

‘The Swedish Disease Act’ (10) obliges HIV-positive persons to disclose information regarding their condition, prior to sexual contact and in connection with medical treatment and care. The Swedish health care system is organized such that all HIV-positive patients are treated and followed at hospital-based outpatient clinics, within departments of infectious diseases, by physicians specializing in infectious diseases and/or venereology.

There is a shortage of studies investigating the differences between HIV-positive Swedish natives and HIV-positive migrants with respect to their exposure to discrimination, stigma, and self-stigma. Intersectional studies on HIV show that ethnicity, gender, class, and nationality have important roles in the exposure to discrimination, stigma, access to health care, and appropriate care among HIV-positive individuals (11, 12). Therefore, this study was designed to describe the experience of HIV-positive migrants during their encounters with the Swedish health care system.

Qualitative research design is essential to provide policy makers and health professionals with the narrative knowledge needed to improve the quality of care and treatment and the access to care (13). It is also important to improve our knowledge of the complexity and correlations between different factors. These include taboos that are linked to HIV, and other factors linked to gender and socioeconomic conditions (9).

Current knowledge about health care and cultural diversity in Sweden is limited. Therefore, this study aims to improve the knowledge of HIV-positive patient experiences within the Swedish health care system to improve the quality of care and treatment.

## Methods

Grounded Theory (14), which is an empirical method, was chosen for generating a deep understanding of the social processes by which people experience their lives and their responses to it. Grounded Theory is a systematic methodology for theory construction using iterative cycles of abductive reasoning through the analysis of data in research areas with limited knowledge. Qualitative interviews were chosen as the data collection method and used in order to get a deeper understanding of the persons studied.

Permission to contact and recruit HIV-positive participants for the study was obtained from the heads of the clinical departments. The participants were seven women and seven men, aged between 29 and 55 years, who had been living in Sweden for 2–20 years (Table 1). The study participants had different backgrounds, education, and socioeconomic status. It was spontaneously disclosed in the discussions when the participants talked about their partners that all except one man identified themselves as heterosexual. The study participants were contacted and recruited from 2011 to 2014 in three outpatient clinics of departments for infectious diseases, in hospitals located in southwest of Sweden.

**Table 1:** Participants’ age, education, and socioeconomic status

	Education			Age (years)			Occupation		Number of years living in Sweden			Country of origin			
					
Participants	Primary school	High school	University	29–39	40–49	50–59	Employed	Unemployed	1–7	7–14	15–20	Africa	South East Asia	South America	Eastern Europe
Women *N*=7	2	5	0	2	3	2	3	4	2	4	3	5	1	1	0
*n* *N*=7	1	5	1	2	4	1	4	3	1	2	2	5	0	1	1

All HIV-positive migrants were given information about the study by the nurses during their visits to the outpatient clinics, together with an informational letter in English or Swedish. Patients who agreed to participate in the study could contact the researcher directly by telephone or through the nurses at the outpatient clinics. The majority of contacts occurred through the clinic nurses.

Sample size and selection of participants were based on theoretical sampling (14), which means that the data were collected and analyzed concurrently. Data were collected using individual, semi-structured, and qualitative interviews. The interview guide was based on the research questions and was reviewed by the co-authors LP and RA. The interviews were led by the first author who is a social worker, with experience in implementing qualitative interviews and working with migrants. This author has worked with HIV prevention at an administrative level in the community. The interviews queried three main themes. How do you perceive your contacts with health services in Sweden? How have you experienced the support provided by the Swedish health care system? What difficulties have you experienced in your contacts with the Swedish health care system? The interview questions provided the framework for the content of the interviews, and also provided opportunities to deepen and extend the discussions.

The participants chose the place of the interview according to their convenience. Eleven participants chose to be interviewed in the outpatient clinic, one in his home, and two at a café. Eleven interviews were conducted in Swedish, two in English, and one with the assistance of a Somali-Swedish translator. The interviews lasted between 40 and 60 min. All interviews were tape-recorded and transcribed verbatim.

Data analysis was conducted in parallel with data collection as described by Charmaz (14). Charmaz's constructivist approach to Grounded Theory considers data collection and analysis as shared experiences and relationships between the researcher and the informant in a specific context.

Three kinds of coding were used during data analysis: open coding, theoretical coding, and constant comparison (14). The first step involved open coding, which identified repeated words or phrases, and axial coding, which identified concepts and their relationships. This process continued until the core category emerged. Then, theoretical coding functioned as the ‘conceptual connector’ to develop relationships between the identified categories and their properties (14). Constant comparison was used during the whole process of data analysis.

Measures were taken to establish trustworthiness of the data and included a thorough description of study design and methods (15). The findings were regularly discussed by the authors. Quotations from the interviews are presented in the text to provide the confirmability of the data.

The Regional Ethical Review Board in Gothenburg granted the ethical approval of this study in 2011 (registration number 681-11). Written informed consent was obtained from each participant before the interview. The tape recorder and the transcribed interviews were kept in a locked, protected place, and only the first author had access. During the interviews, the participants did not show any negative emotional reactions or any other signs that could have indicated an adverse emotional reaction to the questions. If they had displayed an adverse reaction, they would have been referred to the counselors at the clinic.

## Results

**Fig. 1:**
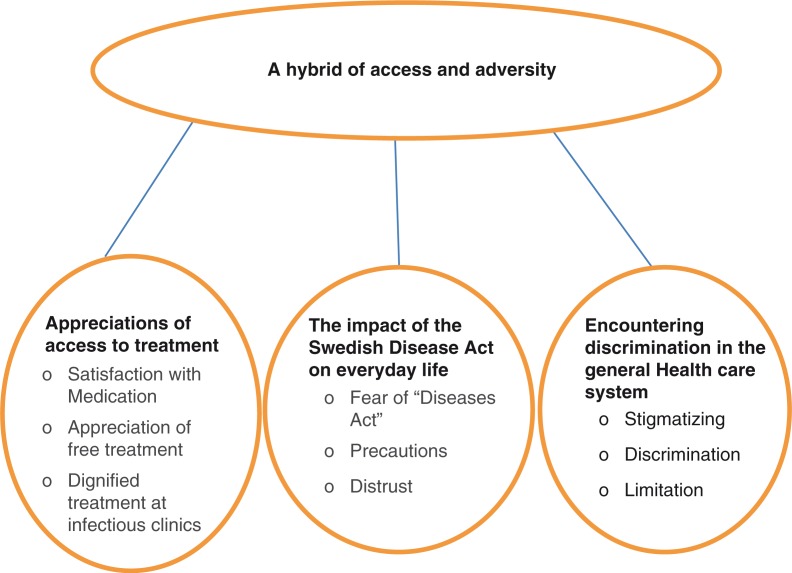
The core category and its three categories.

‘A hybrid of access and adversity’ was identified as the core category of the study. Three additional categories included ‘appreciation of access to treatment’, ‘the impact of the Swedish Disease Act on everyday life’, and ‘encountering discrimination in the general health care system’ (Fig. 1). The core category describes the mixed experiences of the participants within the health care system in Sweden.

### Appreciation of access to treatment

The participants’ appreciation of access to medical treatment was a constant factor in the interviews. In their home countries, the participants had limited access to welfare measures, and they expressed great appreciation of the access to free HIV treatment in Sweden. However, the degree of appreciation and gratitude varied according to the participants’ background and the HIV situation in their home country. Participants who had been diagnosed with HIV before moving to Sweden had greater appreciation of the access to treatment in Sweden than other migrants. An African male participant who was diagnosed with HIV before moving to Sweden expressed his gratitude and appreciation for the free treatment and the positive reception he and his wife had received at the infectious diseases clinic in Sweden in the following statement.

In Africa, sometimes you get medicine that is for pigs. You cannot get treatment without corruption in Africa. In Africa, you never get a letter to go to the hospital, and you only go to the hospital when you are very sick, and in the hospital they don't ask you anything. Here, it is very good. We are very happy because every time we get a letter, we come here on time, get our check-up, and get medicine. In Africa, the doctors think that you disturb them. In Africa, you have to pay 700 US dollars for medicine. I don't have money because I am poor. Here, you can ask about everything and they are happy with you. Every time we get treatment here, we say thanks a lot for this country! I feel like a king here! I am a king and my wife is a queen. This is heaven. (P14)

The participants reported feeling insecure in the health care systems of their home countries because of corruption, lack of medical routines, and the high cost of HIV treatment and care. These factors were the main reasons for participants’ feelings of gratitude and appreciation for the HIV treatment and care they received in Sweden. The infection clinics in Sweden employ advanced medical care and routines, which gave the participants a feeling of security. They reported feelings of confidence in their treatment and their ability to live with their lifelong disease. A female participant expressed her gratitude and confidence regarding her HIV therapy in Sweden in the following statement.

I cannot say anything negative about my situation in Sweden. I have a really good doctor, I have my angels. There are the nurses and they are amazing. (P 4)

The participants expressed a desire to spend more time with the doctors at the infectious diseases clinics where they received their regular treatment, to express their concerns and fears and ask questions about their disease and its treatment. They described feelings of trust toward these doctors, and they wanted more time to talk with them. For most participants, the contact with health care professionals at the outpatient clinics was their only social contact with the Swedish society. For many participants, the infectious diseases clinics were the only places where they allowed themselves to be open about their HIV infection without fear or worry. For these reasons, they felt a strong need to communicate with their physicians about their concerns and get updated information about their prognosis and other aspects of the disease.

### Impact of the Swedish Disease Act on everyday life

The unmarried participants in this study described the Swedish Disease Act as an obstacle to their happiness. It made their struggle to find love and a partner to combat loneliness more difficult. One of the male participants described the effect of the act on his life as follows.

It is crazy … some things you understand, but other things you do not understand. Even if you tell your partner that you are HIV positive, your partner can sue you later. So if she or he says that you have not told her or him, it does not matter even if you have said it, you will be punished anyway. So it is really about two people, that they should both take responsibility for protecting themselves. If you are forced to tell your HIV status to every prospective partner, it is difficult if you are a migrant. Then, the risk is that you tell a Swedish girl or girlfriend, and she tells it to the next person, and then they say bad things about migrants. (P5)

Participants’ responses during the interview indicated that the requirement to provide sexual partners with information about their HIV status increased their already vulnerable situation. They felt that their vulnerability increased both in general and in sexual relationships, and reported that they experienced feeling a loss of power and control. Participants felt that a lack of confidence, which may already exist at the beginning of sexual relationships, was likely to become more difficult to deal with because of the Swedish Disease Act. The HIV-positive migrants described their experiences in relationships as having less power, being unequal, and being discriminated against by the partner. The participants also stated that they might be discriminated against by the court in cases of legal conflicts with sexual partners, a risk that caused feelings of fear and uncertainty. They believed that even if they did perform the required disclosure of their HIV status to sexual partners, they would always be considered guilty in legal cases.

The participants experienced several difficulties related to the requirement to provide sexual partners with information about their HIV status. Some argued that it was impossible to talk about their HIV status to migrant partners because of the oppressive taboos associated with HIV in their home countries. The one male participant who was identified as homosexual expressed less concern regarding the disclosure of his HIV status to Swedish partners, whereas heterosexual participants expressed more concern regarding the disclosure of their HIV status to Swedish partners.

In general, the required HIV disclosure to sexual partners was perceived negatively, making it more difficult to find a new partner and to continue an existing relationship. A few participants stated that they had chosen abstinence instead of seeking sexual contacts because of the difficulties. However, most participants described searching for a sexual partner who also was HIV positive, although this also was perceived as difficult because of the fear of being identified as HIV positive.

### Encountering discrimination in the general health care system

The participants’ encounters with general health care units outside of the infectious diseases clinics were described as discriminatory. This included contacts with other health care centers, dental care, and other specialist care facilities. One participant described her experience with health care professionals at a mammography clinic after they heard she was HIV-positive.

I went to have a mammography test a few months ago. When they asked me if I was healthy, I said that I had a blood disease. The nurses asked, ‘What blood disease do you have? HIV’ I said yes. After that, it was frighteningly quiet in the room, and everyone started getting stiff. While I was sitting in the room, I saw how they picked up everything and started cleaning the room, instruments, and equipment. If I had not told them that I am HIV positive, they would have just picked up the exam paper and put a fresh paper. You feel that they begin to clean up even while you are still sitting there. It crushes the heart. (P10)

Discrimination during encounters with health care professionals was described as indirect and subtle, and was expressed by means of body language, looks, and surprised reactions right after they were informed about the participants’ HIV status. For example, the staff started cleaning up medical equipment in front of the participants as soon as they were informed. The participants were referred to specially adapted HIV units with specialist staff for some forms of treatment and care, but experienced difficulties accessing some of them, such as special units for dental and prenatal care. Participants perceived this as discrimination because they did not have equal access to general health care as other patients and had to wait longer to receive care at the special HIV care units.

Another criticism that participants expressed was that they had to go back and forth between different specialist units for treatment. Although these special units were adapted to care for and treat HIV-positive patients, each unit had a restricted list of specialist services. For example, a specialist unit would perform blood tests, forcing the patients to return to the infectious diseases outpatient clinic for blood tests. This was the case at the prenatal care unit specially adapted for pregnant patients who were HIV positive.

### A hybrid of access and adversity

‘A hybrid of access and adversity’ is the core category that describes the participants’ experiences within the Swedish health care system. The terms ‘access and adversity’ describe the tension between the access to care offered by the Swedish health care system, contrasted with its limitations for HIV patients. These include the participants’ experiences of insufficient psychosocial support and difficulties in their encounters with the Swedish health care system.

The Swedish welfare system provides free health care and access to free antiviral therapy, which is highly appreciated by the participants. However, the participants also described their struggle with the requirement to always inform sexual partners about their HIV status, which is mandatory under the Swedish Disease Act (10). The participants’ experiences were grouped into two categories.

1. Limitations imposed by certain regulations in the Swedish health care system governing HIV-positive patients. The impact of these limitations on the participants’ everyday lives was described as significant and frustrating. The limitations also were closely associated with the participants’ perceptions of discrimination.
2. Limitations caused by the attitudes toward, or fear of, HIV infection among general health care professionals outside of the infectious diseases clinics. Participants experienced these attitudes not only as discriminatory but also as obstacles that limited their contacts with the general health care system.

The participants’ descriptions of their needs for social support were diverse. However, the most common need expressed by the participants was for more time to talk with their doctors at the clinics. The participants felt that it was very important to receive detailed current information about HIV from the doctors. This communication with the doctors helped them to feel safe and also helped them trust in their own abilities to cope with lifelong chronic disease.

Participants’ descriptions about what they needed from the health care system varied depending on gender, age, living conditions, and marital status. For example, young women felt that they did not have access to the appropriate fertility treatment when they had difficulties in getting pregnant. Participants who did not have a sexual partner experienced more difficulties because of the obligation to disclose their HIV status under the Swedish Disease Act.

## Discussion

The results of this study show that, in spite of access to free health care and free antiviral therapy, HIV-positive migrants in Sweden experience some limitations in the provision of general health care. The participants’ appreciation of free access to antiviral therapy was combined with their negative experiences of what they perceived as legal limitations on their behavior and discrimination in the general health care services. The Swedish Health and Medical Services Act (16) mandates that all citizens and residents in Sweden should have equal access to health care regardless of gender; socioeconomic status; geographical region of residence; or national, ethnic, cultural, religious, and linguistic background. The migrants’ origins and home countries are a significant factor in how they experience care and treatment in the country of migration (17). The participants’ appreciation of free access to health care in Sweden could be related to their earlier experience of limited access to health care in their home countries.

Swedish health care policy emphasizes equal access to necessary health care, which meets the individual's needs, and the importance of patient participation in treatment, which emphasizes civic involvement and equal treatment for everyone (16). An issue of concern for the participants is the experience of discrimination and stigma they claim to have experienced in the general health care services outside of the infectious diseases clinics. Experiences of discrimination from general health care providers may reflect a fear of HIV among these workers due to the fact that they have received less training and are less familiar with standardized procedures for working with HIV-positive patients. These workers also may lack knowledge about the routes of viral transmission of this disease. Thus, it is important to address this fear with increased information, training, and support.

Although migrants may be legally entitled to access to health care in Europe, these legal requirements are not always well-known or respected in practice (18). The right to health contains four essential elements (19): availability, accessibility, acceptability, and quality. The results of this study show that the HIV-positive migrants experience limited availability of care for important co-morbidity parameters at the infectious diseases clinics and limited quality of care within the general health care system.

The experience of stigma among HIV-positive individuals is general and is connected with the fear of disclosure of their HIV status, and the anxiety about losing a job, partner, home, and family (20). A qualitative study reported that an important requirement for appropriate psychosocial support for HIV-positive African women in England is that it is ‘non-judgmental’ and ‘personalized’ (21). This study shows that participants felt that the obligation to disclose their HIV status to prospective sexual partners, which is mandatory under The Swedish Disease Act (10), made their lives more difficult. They considered that it complicated their already vulnerable living condition. This increased their feelings of loneliness and stigmatization.

There was an observed gender perspective in the participants’ experiences of difficulty under The Swedish Disease Act. Unmarried male participants expressed more concern about finding a sexual partner, whereas female participants expressed more concern related to pregnancy and childbirth. There is free access to fertility treatment for women up to a certain age in the Swedish health care system. However, for HIV-positive women, the free access is limited to insemination. If a woman does not respond to insemination, then she is denied access to advanced fertility treatment that is available to HIV-negative women. The homosexual participant who had moved to Sweden due to marriage, experienced less difficulty with The Swedish Disease Act.

According to a recent statement from the Public Health Agency of Sweden and the Swedish Reference Group for antiviral therapy (22), effective antiviral therapy reduces the risk of patient transmission of HIV to approximately zero and essentially eliminates the spread of the infection at the population level. Many studies have confirmed that social support reduces stigma and increases openness about HIV, which in turn improves the living conditions of infected individuals. A meta-analysis identified a positive correlation between a willingness to disclose HIV status and social support systems, and a negative correlation between social stigma and disclosure (23). Further studies are needed to identify whether experiences of discrimination, fear, and social isolation substantially differ between HIV-positive migrants and HIV-positive Swedes.

## Conclusions

The findings of this study demonstrate that free access to health care and antiviral therapy is necessary but not sufficient for HIV-positive migrants to feel satisfied with the Swedish health care system. Limitations in general health care access and perceptions of discrimination from general health care workers are the main reasons for dissatisfaction among HIV-positive migrants. These limitations and social barriers also influence the everyday lives of HIV-positive migrants.

Although more studies are needed within this field, the findings of this study should be taken into consideration by health care professionals and policy makers within the Swedish health care system. Currently, there is a need to reduce the discrimination in general health care services and to optimize the social support system and social network of this vulnerable group. These actions will improve the diverse needs of HIV-positive migrants within the Swedish health care system and Swedish society.

## Paper context

Many studies show that late testing, access to care and treatment, and the ability to remain in long-term care challenge the effective delivery of HIV-related care to the migrants globally. The main finding of this qualitative study is that HIV-positive migrants experience discrimination in the Swedish general health care system outside the infectious disease clinics. There is a need to optimize care, social support system, and social network of this vulnerable group.
